# Spatial and Temporal Analysis of Alphavirus Replication and Assembly in Mammalian and Mosquito Cells

**DOI:** 10.1128/mBio.02294-16

**Published:** 2017-02-14

**Authors:** Joyce Jose, Aaron B. Taylor, Richard J. Kuhn

**Affiliations:** a Department of Biological Sciences, Purdue University, West Lafayette, Indiana, USA; b Bindley Bioscience Center, Purdue University, West Lafayette, Indiana, USA; c Purdue Institute of Inflammation, Immunology and Infectious Disease, Purdue University, West Lafayette, Indiana, USA; University of Pittsburgh School of Medicine

## Abstract

Sindbis virus (SINV [genus *Alphavirus*, family *Togaviridae*]) is an enveloped, mosquito-borne virus. Alphaviruses cause cytolytic infections in mammalian cells while establishing noncytopathic, persistent infections in mosquito cells. Mosquito vector adaptation of alphaviruses is a major factor in the transmission of epidemic strains of alphaviruses. Though extensive studies have been performed on infected mammalian cells, the morphological and structural elements of alphavirus replication and assembly remain poorly understood in mosquito cells. Here we used high-resolution live-cell imaging coupled with single-particle tracking and electron microscopy analyses to delineate steps in the alphavirus life cycle in both the mammalian host cell and insect vector cells. Use of dually labeled SINV in conjunction with cellular stains enabled us to simultaneously determine the spatial and temporal differences of alphavirus replication complexes (RCs) in mammalian and insect cells. We found that the nonstructural viral proteins and viral RNA in RCs exhibit distinct spatial organization in mosquito cytopathic vacuoles compared to replication organelles from mammalian cells. We show that SINV exploits filopodial extensions for virus dissemination in both cell types. Additionally, we propose a novel mechanism for replication complex formation around glycoprotein-containing vesicles in mosquito cells that produced internally released particles that were seen budding from the vesicles by live imaging. Finally, by characterizing mosquito cell lines that were persistently infected with fluorescent virus, we show that the replication and assembly machinery are highly modified, and this allows continuous production of alphaviruses at reduced levels.

## IMPORTANCE

Reemerging mosquito-borne alphaviruses cause serious human epidemics worldwide. Several structural and imaging studies have helped to define the life cycle of alphaviruses in mammalian cells, but the mode of virus replication and assembly in the invertebrate vector and mechanisms producing two disease outcomes in two types of cells are yet to be identified. Using transmission electron microscopy and live-cell imaging with dual fluorescent protein-tagged SINV, we show that while insect and mammalian cells display similarities in entry and exit, they present distinct spatial and temporal organizations in virus replication and assembly. By characterizing acutely and persistently infected cells, we provide new insights into alphavirus replication and assembly in two distinct hosts, resulting in high-titer virus production in mammalian cells and continuous virus production at reduced levels in mosquito cells—presumably a prerequisite for alphavirus maintenance in nature.

## INTRODUCTION

Alphaviruses are maintained in nature by transmission between susceptible vertebrate hosts by hematophagous arthropods, and they multiply in the tissues of both their vertebrate and invertebrate hosts. Infection of cultured vertebrate cells is normally cytopathic, whereas infection of cultured mosquito cells results in a noncytopathic, persistent infection (1). Members of the New World encephalitic alphaviruses (Venezuelan and eastern and western equine encephalitis viruses) cause encephalitis in human and domestic animals. Infections from Old World arthritogenic alphaviruses, such as Ross River virus and chikungunya virus (CHIKV), lead to debilitating musculoskeletal disease in humans, whereas the other family members cause mild infections, including a self-limiting febrile illness characterized by fever, skin rashes, and arthritis (2). Recent CHIKV outbreaks have resulted in millions of cases in nearly 40 countries (3). Currently, there are no antivirals or vaccines available to control these infections.

Alphaviruses, like other positive-strand RNA viruses, replicate their 11.7-kb RNA genome in association with cellular membranes (4, 5). Nonstructural proteins nsP1, nsP2, nsP3, and nsP4 are translated as polyproteins P123 and P1234 from the 5′ two-thirds of the 49S genomic RNA. These polyproteins are proteolytically processed by nsP2 and along with the viral RNA and host proteins form the membrane-bound replication complex (RC) that is required for genome replication and transcription of subgenomic RNA. Virus replication induces bulb-shaped membrane invaginations with a diameter of ~50 nm called “spherules,” where viral RNA synthesis occurs. Spherules line the limiting membranes of large (0.6 to 2.0 μm in diameter) complex cytoplasmic vacuolar structures type I (CPV-I) (4). CPV-I likely originate from endosomes and modified secondary lysosomes (6). Spherules are also located at the plasma membrane (PM) and are presumably guided there by nsP1, as it has been shown to have an affinity for lipids specific for the cytoplasmic leaflet of the PM. These RCs from the PM subsequently are endocytosed to form CPV-I (7, 8). CPV-I has been proposed to be the site of viral RNA synthesis, with viral nsPs accumulating at the cytoplasmic neck of the spherules and the newly synthesized RNA diffusing into the cytoplasm through the pore of the spherule (6).

The structural proteins CP (capsid protein), E3, E2, 6K, and El are translated from a 26S subgenomic RNA that corresponds to the 3′ third of the genome. The structural polyprotein is autocatalytically processed at the N terminus by the capsid protease, and CP is released into the cytoplasm. The envelope glycoproteins are transported through the secretory pathway and are processed by the host signalase in the endoplasmic reticulum (ER) and furin in the late Golgi complex, respectively. In the cytoplasm, 240 copies of CPs encapsidate a single genomic RNA to form the nucleocapsid core (NC) that binds the glycoprotein spikes present on the PM during virus budding. The type II cytopathic vacuole (CPV-II) is the predominant virus-induced vacuolar structure that contains the E1/E2 glycoproteins and numerous NCs attached to its cytoplasmic side. CPV-II originates from the *trans*-Golgi network ~4 h postinfection (p.i.) (9). Electron tomography studies have revealed that the E1/E2 glycoproteins are arranged in a helical array within CPV-II in a manner that resembles their organization on the viral envelope (10). CPV-II is presumably involved in the intracellular transport of the glycoproteins from the *trans*-Golgi network to the PM and also transport of NCs to the site of virus budding (10). The specific interaction of the cytoplasmic domain of glycoprotein E2 with the hydrophobic pocket present on the CP promotes Sindbis virus (SINV) budding from the PM.

A variety of techniques, including live imaging and transmission electron microscopy (TEM), have been used to derive structural and morphological characterizations of alphaviruses in mammalian cells, but the mechanisms of replication and assembly remain poorly understood in the infected cells of the mosquito vector. Live-cell imaging provides a powerful means to probe the mechanisms by which viruses are transported between cells and to explore the spatial and temporal dynamics of viral assembly and egress. Fluorescent protein (FP)-tagged SINV with insertions in replication proteins nsP2, and nsP3 and dually labeled viruses with green fluorescent protein (GFP) in nsP2 and mCherry in nsP3 have been used extensively to study RC formation and host protein interactions (11, 12). Live-cell imaging coupled with TEM has demonstrated the generation of CPV-I in cells infected with Semliki Forest virus (8). We and others have described FP-tagged E2 for analyses of virus entry and budding (13–15). In this study, we established a dual-color, live-cell imaging platform that enabled the simultaneous monitoring of alphavirus replication and assembly in insect and mammalian cells. We used live-cell imaging of fluorescently labeled nonstructural and structural proteins to characterize viral replication and assembly in mosquito and mammalian cells. By means of TEM and live-cell imaging using dually labeled viruses, we demonstrate that RCs in mosquito cells form around acidic vesicles that contain the viral glycoproteins. However, in mammalian cells, the double-stranded RNA (dsRNA)-containing RCs do not associate with glycoproteins. The morphologies of these cytopathic vacuoles as characterized by TEM were found to differ in the insect vector and mammalian host cells, and we provide evidence for the biogenesis of internal budding in insect cells. We generated and characterized mosquito cell lines persistently infected with wild-type (WT) and FP-tagged SINV that allowed us to identify morphological and structural differences and compared the growth kinetics of acute versus persistent infections. Furthermore, we provide evidence for exclusive internal budding in persistently infected mosquito cells and reduced glycoprotein expression at the PM. These studies suggest that there are fundamental differences between the interaction of viral replication and structural components with cells of the insect vector and those of a mammalian host that suggest mechanistic differences. Insights from this study relating the spatial and temporal dynamics of virus replication and assembly in mammalian and mosquito cells demonstrate the fundamental processes by which viruses exploit the resources and environment of their specific hosts.

## RESULTS

### Construction and characterization of dually FP-tagged SINV.

**FIG 1:**
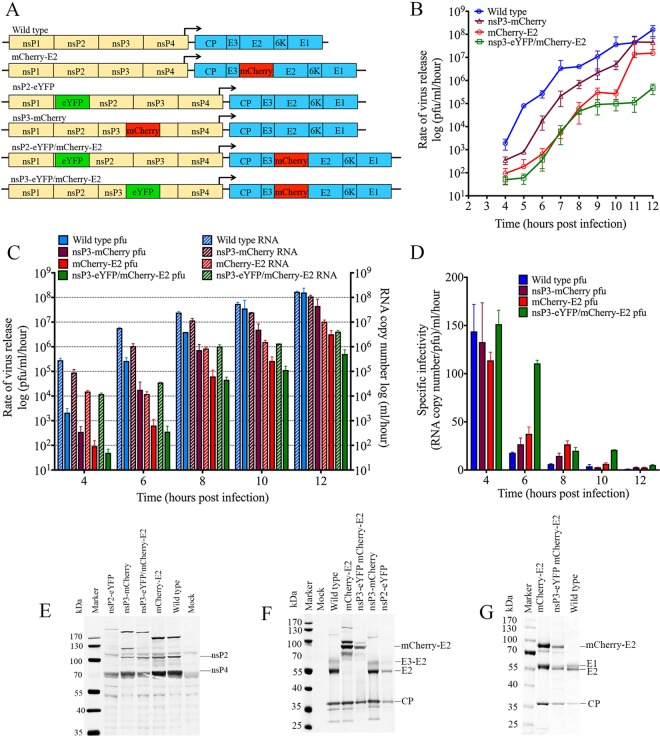
Construction and characterization of FP-tagged viruses. (A) FP-tagged SINV constructs generated for the study. Sequences encoding the fluorescent proteins eYFP and mCherry were cloned into pToto64, a cDNA clone of SINV, as fusion proteins of nsP2, nsP3, or E2. (B) One-step growth curve analysis of FP-tagged viruses from BHK cells. BHK cells were infected with WT or FP-tagged viruses at an MOI of 5, medium was changed every hour for 12 h, and the rate of virus release (PFU per milliliter per hour) was determined using standard plaque assays. (C) Quantitation of the number of virus particles released into the medium for WT and FP-tagged viruses at 4, 6, 8, 10 and 12 h p.i. The total number of genome RNA molecules was determined by qRT-PCR using a standard curve of a known amount of *in vitro-*transcribed SINV RNA molecules. The PFU in these samples were determined by standard plaque assays of the virus supernatant used collected at 4, 6, 8, 10, and 12 h p.i. from infected BHK cells. (D) Specific infectivity (particle/PFU ratio) of FP-tagged virus calculated from panel C. (E) Western analysis of the cytoplasmic extracts of FP-tagged SINV-infected BHK cell lysates to detect nonstructural protein processing. The blot was probed with anti-nsP2 and anti-nsP4 rabbit polyclonal antibodies. (F) Western analysis of the cytoplasmic extracts of FP-tagged SINV-infected BHK cell lysates. The blot was probed with anti-CP and anti-E2 rabbit polyclonal antibodies. (G) SDS-PAGE analysis of purified FP-tagged and WT virus showing the mCherry protein tagged to the E2 protein.

FP-tagged SINV constructs have proved to be useful tools to detect RCs from infected cells and to study virus entry and budding (11, 14, 15). Sites for nsP2, nsP3 (11, 12, 16), and E2 cloning were previously described (13, 14, 17). Monomeric fluorescent proteins mCherry and enhanced yellow fluorescent protein (eYFP) were selected for cloning experiments. Along with single tags at nsP2, nsP3, and E2, dually labeled constructs were generated with nsP2-eYFP/mCherry-E2 and nsP3-eYFP/mCherry-E2 to simultaneously study replication and assembly (Fig. 1A). Dually labeled nsP2 and nsP3 have been previously used (11), demonstrating that genome replication and packaging of the tagged viruses were not affected by these insertions. One-step growth kinetic analyses of WT, mCherry-E2, mCherry-nsP3, and nsP3-eYFP/mCherry-E2 dually labeled viruses were performed (Fig. 1B). All tagged viruses were reduced by at least 1 log and up to 3 logs in virus yield compared to WT virus (Fig. 1B). Of the two dually labeled viruses, nsP2-eYFP/mCherry-E2 produced small plaques with a 4-log reduction in virus titer and was not subjected to further growth kinetic analyses owing to the low titer (4 × 10^4^ PFU/ml). Although reduced in PFU, this virus grew adequately enough for live-cell imaging and allowed us to compare the localization of nsP2-eYFP/mCherry-E2 with that of nsP3-eYFP/mCherry-E2 in dually labeled virus-infected cells. To determine whether the reduction in virus production for the FP-tagged viruses observed in growth kinetic analysis was due to reduced specific infectivity, Quantitative real-time reverse transcription-PCR (qRT-PCR) analysis of the number of RNA molecules released into the medium was performed (Fig. 1C) at five different time points. This showed a consistent reduction in the number of RNA molecules released into the medium compared to WT virus akin to the reduction in PFU. Based on the calculated particle/PFU ratio at each time point (Fig. 1D), the specific infectivity of most of the FP-tagged viruses was found to be comparable to that of WT SINV. The polyprotein processing of nonstructural (Fig. 1E) and structural proteins (Fig. 1F) of these tagged viruses was monitored by Western blotting analyses of cytoplasmic extracts from infected cells using combinations of antisera against nsP2, nsP4, CP, and E2. SDS-PAGE analysis showed the incorporation of mCherry-E2 into the virions in the dually labeled virus similar to that of mCherry-E2 (Fig. 1G).

### Comparison of RNA replication and growth kinetics of C6/36 and BHK cells using WT and mCherry-E2 virus.

**FIG 2:**
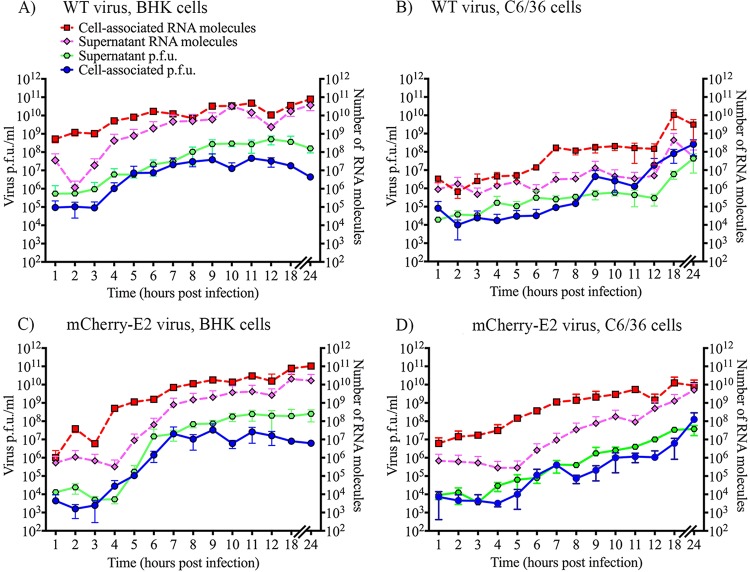
Replication and growth kinetic analyses of BHK and C6/36 cells infected with WT and mCherry-E2 viruses. Medium over 10^6^ cells or lysates of 10^6^ cells were used for the analysis. Virus-infected cells were lysed with repeated freeze-thaw cycles to recover cell-associated virus, and the numbers of infectious particles in the supernatant and cell-associated viruses were determined by standard plaque assays. Total RNA was extracted from 10^6^ infected cells and culture supernatants, and the total number of viral genome RNA molecules present in the extracts was quantified using qRT-PCR. The number of RNA molecules from virus culture supernatant or cytoplasmic extracts and the PFU associated with the cells and supernatants were plotted for the WT from BHK cells (A), WT from C6/36 cells (B), mCherry-E2 from BHK cells (C), and mCherry-E2 from C6/36 cells (D).

Virus replication occurs at a lower rate in mosquito compared to mammalian cells (Fig. 2). SINV also establishes a persistent infection in mosquito cells, whereas mammalian cells experience acute cytopathic infection that results in cell death in ~24 h. Experiments were performed to explore why BHK and C6/36 cells exhibit fundamental differences in the rates of virus replication and budding and how spatial differences of RCs on cytopathic vacuoles affect virus assembly and budding. Time course experiments were conducted using qRT-PCR of total cytoplasmic RNA and genome RNA from released virus particles at each hour for 12 h and then at 18 and 24 h p.i. (Fig. 2). Plaque assays of virus particles collected from supernatants and cell lysates were also performed at these time points. Plaque assays to detect the number of cell-associated virus particles from mosquito cells were performed to determine the rate of internal budding and the time postinfection when the greatest number of infectious particles accumulated in infected cells.

Analyses of virus growth and RNA replication kinetics in BHK cells showed that virus replication and release significantly increased at 6 and 9 h p.i., respectively (Fig. 2A and B), after which there were ~10 internalized virus particles (either internal budding or virus internalization by entry) in every cell (in a total of 10^6^ cells [Fig. 2]). The number of virus particles in the supernatant was significantly greater than the number of cell-associated infectious virus particles per cell. The total number of RNA molecules in the cell represents the sum of replicating RNA and RNA packaged into cytoplasmic cores, and this number constantly exceeded the number of RNA molecules outside the cell, suggesting that only a fraction of RNA/cores bud, which is consistent with previous reports (17).

In mosquito cells, virus replication significantly increased at 7 h p.i., followed by a spike in the release of virus at 8 h p.i. After this, the numbers of RNA molecules and PFU associated with the cells were higher than the RNA molecules or PFU contained in the supernatant. The number of released virus particles peaked at 18 h p.i., at which time there were ~100 infectious virus particles in each mosquito cell (Fig. 2C). A comparable trend was observed in the replication and assembly of mCherry-E2-tagged virus compared to wild-type virus (Fig. 2A to D). Tagged virus exhibited a 2-h delay in maximal replication and virus release compared to WT virus (Fig. 2A and C) in BHK cells.

### BHK and C6/36 cells show spatial differences in the distribution of replication complexes.

**FIG 3:**
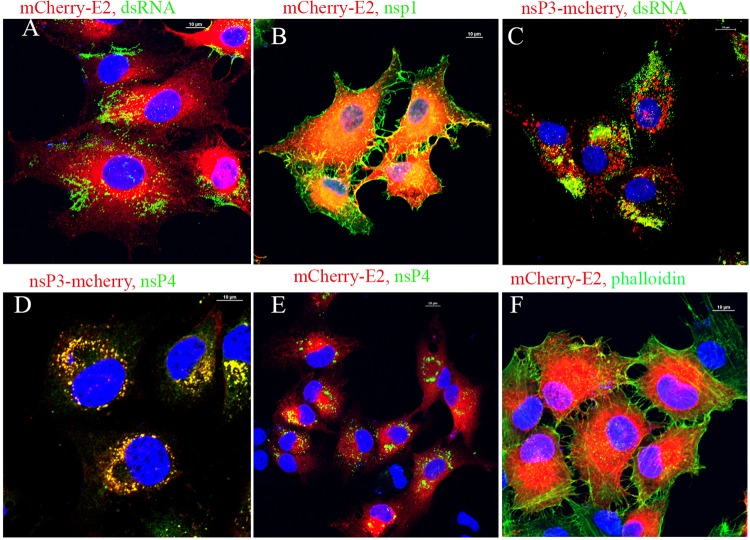
IF analysis of BHK-15 cells infected with FP-tagged viruses showing the distribution of viral replication complexes and glycoprotein E2. Cells infected with FP-tagged SINV were subjected to IF analysis at 6 h p.i. using antibodies against dsRNA (A and C), nsP1 (B), or nsP4 (D and E), as indicated in the figure. The filamentous actin was detected using phalloidin (F).

BHK cells infected with WT or FP-tagged SINV were subjected to immunofluorescence (IF) analyses at 6 h postinfection using antibodies against double-stranded RNA (dsRNA), nsP1, and nsP4 (Fig. 3). Filamentous actin was detected using phalloidin. Alphavirus RCs in infected BHK cells, verified by the presence of dsRNA, were distributed throughout the cytoplasm (Fig. 3A and C). RCs were detected at internal vesicles (presumably endolysosomes) and PM but not on filopodial extensions (Fig. 3A, C, D, E). Replication proteins nsP3, and nsP4 showed a very strong colocalization with internal vesicles (CPV-I) along with dsRNA (Fig. 3C to E). Unlike most of the replication proteins, nsP1 was present on filopodial extensions (Fig. 3B). The filopodial extensions contained filamentous actin that colocalized with nsP1 (Fig. 3F).

**FIG 4:**
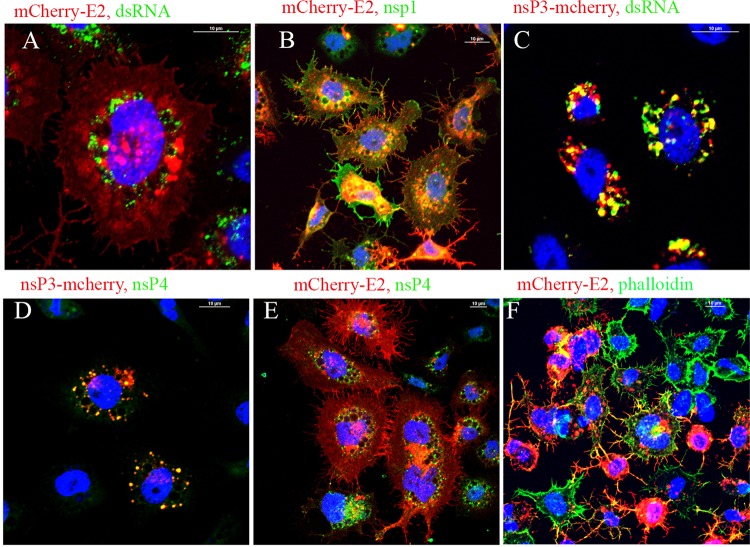
IF analysis of C6/36 cells infected with FP-tagged viruses. C6/36 cells were infected with FP-tagged viruses and were subjected to IF analysis at 12 h p.i. using antibodies against dsRNA (A and C), nsP1 (B), or nsP4 (D and E), as indicated in the figure. The filamentous actin was detected using phalloidin (F).

In C6/36 cells infected with nsP3-eYFP or mCherry-E2 virus, the replication proteins nsP3 and nsP4 localized to the outer surface of the mCherry-E2-containing red vesicles (Fig. 4C and D). In C6/36 cells, replication proteins were distributed around the large cytopathic vesicles (Fig. 4A, C, D, and E) that contain E2. Specifically, the nonstructural proteins and dsRNA were located on the outer membrane of these vesicles. Similar to that shown for BHK cells, nsP1 colocalized with the filopodial extensions at the PM of C6/36 cells (Fig. 4B) and also with filamentous actin (Fig. 4F). Similar to BHK cells, the dsRNA and replication proteins nsP2, nsP3, and nsP4 were not located on the filopodial extensions, but in contrast to BHK cells, these were not present on the PM (Fig. 4A, C, D, and E).

### TEM analysis of BHK and C6/36 cells infected with WT SINV.

**FIG 5:**
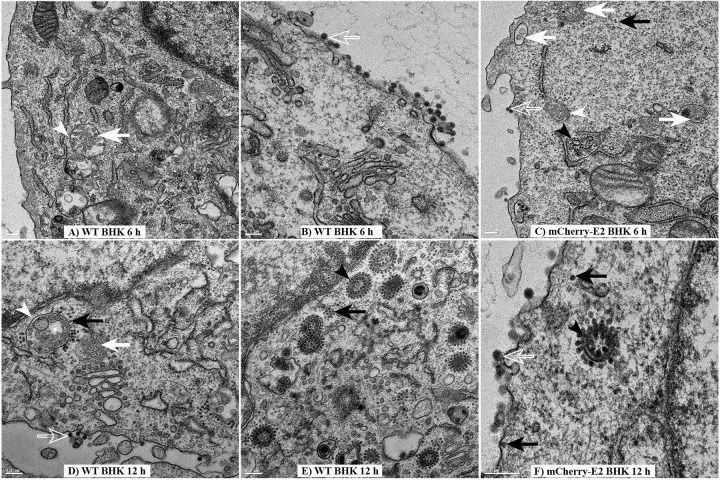
TEM analyses of infected BHK cells. Shown are the results of TEM analysis of BHK cells infected with WT and mCherry-E2 virus. BHK cells were infected with WT (A, B, D, and E) or mCherry-E2 (C and F) virus at an MOI of 5 and fixed for TEM analysis at 6 h (A to C) and 12 h (D to F) p.i. Budding viruses (open white arrows), NCs (solid black arrows), and replication spherules (solid white arrows) are marked. Solid white arrowheads indicate CPV-I, and solid black arrowheads indicate CPV-II. Scale bars represent 200 nm. CPV-I does not contain any internally budded particles (A and E).

Thin-section electron microscopy of BHK cells infected with WT SINV (Fig. 5A, B, C, D, and E) and mCherry-E2 (Fig. 5C and F) at 6 h p.i. (Fig. 5A to C) and 12 h p.i. (Fig. 5D and E) showed the presence of NCs, replication spherules, and budding virions. CPV-I and CPV-II were both present in the infected cells at 6 h p.i., with CPV-I larger and more abundant than CPV-II (Fig. 5A and C). The inner membrane of CPV-I was associated with small replication spherules that possibly contained replicating RNA. The spherules were connected to the cytoplasmic side where the NCs were present (Fig. 5D). A few spherules were also observed on the PM (Fig. 5C) that appeared to be endocytosed. CPV-I was also seen opposing the rough endoplasmic reticulum (Fig. 5C and D). CPV-II are observed at both 6 h and 12 h p.i. Different types of CPV-II were seen associated with NCs on the cytoplasmic side of the vacuole as well as the interior of the double-membrane CPV-II (Fig. 5E and F). These were different from the endocytic vesicles since endocytic vesicles lack NC on the cytoplasmic side of the membrane. Virus budding occurred predominantly from the PM (Fig. 5B to D and F). Although budded particles were observed to associate with the PM, some of these virus particles seemed to be endocytosed (Fig. 5D). Compared to 6 h p.i., CPV-II were more abundant at 12 h p.i. (Fig. 5E). No accumulation of internally budded particles was observed in BHK cells infected with SINV.

**FIG 6:**
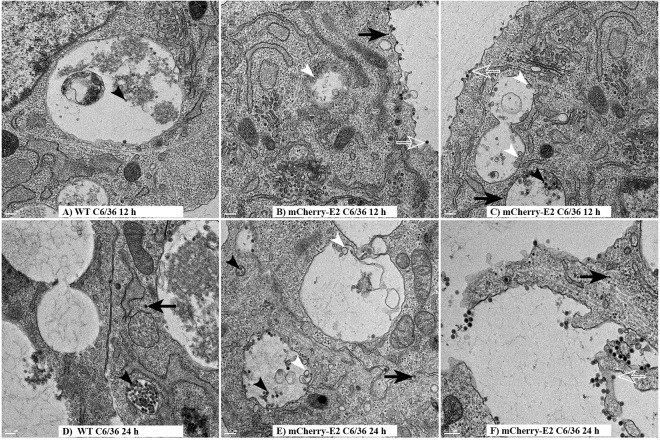
TEM analysis of mosquito cells infected with WT and mCherry-E2 virus. C6/36 cells were infected with WT (A and D) or mCherry-E2 virus (B, C, E, and F) at an MOI of 5 and fixed for TEM analysis at 12 h (A to C) and 24 h (D and E) p.i. Budding viruses (open white arrows), NCs (solid black arrows), and replication spherules (solid white arrows) are shown. Solid black arrowheads point to internally budded virions. Scale bars represent 200 nm. Large cytopathic vacuoles (diameter of 0.5 to 2 μm) containing replication spherules and internally budded virus particles (A and D) are seen. NCs are also seen at the cytoplasmic side of the vesicles.

Mosquito cells (C6/36) infected with WT SINV (Fig. 6A and D) and mCherry-E2 (Fig. 6B, C, E, and F) were examined by thin-section TEM at 12 h p.i. (Fig. 6A to C) and 24 h p.i. (Fig. 6D and E). Similar to what was observed in BHK cells, virus budding, budded virions, NCs, and replication spherules were identified in the infected mosquito cells. Classical CPV-I and CPV-II were not found in alphavirus-infected mosquito cells. Nevertheless, throughout infection, large cytopathic vacuoles (0.5 to 2 μm in diameter) with properties intermediary to CPV-I and CPV-II were present that contained replication spherules and internally budded virus particles (Fig. 6A and D). Even though we observed large numbers of spherules on the inner membranes of vacuolar structures, in all the cells analyzed by TEM, spherules were never observed on the PM of C6/36 cells. NCs involved in virus budding were observed at the PM as well as on the cytoplasmic side of the large vacuoles (Fig. 6E) that are involved in internal budding. The internally budded virus particles eventually were secreted as individual virions, presumably aided by the secretory pathway (Fig. 6B and F). Glycoprotein spikes present on the PM envelope, the cytoplasmic NC, and virus is subsequently released from the cell surface (Fig. 6B, C, and F). Detailed analysis of the different types of cytopathic vacuoles present in the SINV-infected C6/36 cells (see Fig. S1 in the supplemental material) revealed that the replication spherules resided on the inner membrane of the cytopathic vacuoles, similar to the CPV-I of BHK cells. Additionally, internally budded virus particles were seen inside the same vacuoles (Fig. 6C and E). Intraluminal vesicles (Fig. 6A, C, and E) and budded viruses were seen in some vacuoles. Rough endoplasmic reticulum and Golgi complexes were also seen in close proximity to these vacuoles (Fig. 6A, C, D, and E).

10.1128/mBio.02294-16.1 FIG S1  Types of cytopathic vacuoles present in the SINV-infected BHK (CPV-I and CPV-II) cells and C6/36 cells. (A to D) CPV-I associated with small replication spherules (Sp) on its inner membrane, which is connected to the cytoplasm with an opening at the spherule-neck region. Nucleocapsid cores (NCs) and rough endoplasmic reticulum (RER) are present in close proximity to CPV-I. (B) Single-membrane CPV-II with a lipid bilayer seen associated with NCs on the cytoplasmic side of the vacuole. (C) Single-membrane CPV-II (one lipid bilayer) with internally budded virus particles (Vi) and cytoplasmic nucleocapsid cores. (D) Double-membrane CPV-II (two lipid bilayers) with NCs attached to the inner and outer lipid bilayers and seen associated with filamentous structures in the cytoplasm. The scale bars represent 200 nm. (E to H) Types of cytopathic vacuoles present in SINV-infected mosquito cells. (E) Replication spherules (Sp) are present inside the cytopathic vacuoles similar to the CPV-I of BHK cells. There are also internally budded virus particles seen inside the vacuoles. (F) NCs are seen on the cytoplasmic side associated with the vacuoles. (G) Intraluminal vesicles (ILV) and budded viruses (Vi) are seen in some vacuoles. RER and the Golgi complex are in close proximity to the vacuole. (H) A large accumulation of internally budded virions is seen inside the mosquito cells. The scale bars represent 200 nm. Download FIG S1, TIF file, 5 MB. Copyright © 2017 Jose et al. 2017 Jose et al. This content is distributed under the terms of the Creative Commons Attribution 4.0 International license.

### Live-cell imaging.

**FIG 7:**
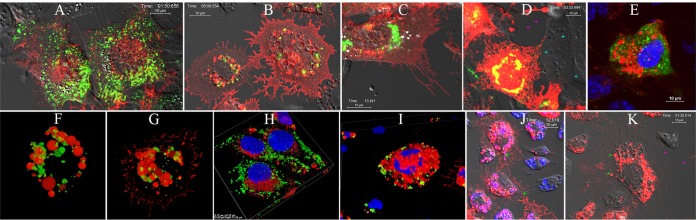
Live imaging of FP-tagged viruses. (A) BHK cells infected with nsP3-eYFP/mCherry-E2 dually labeled virus showing the distribution of replication proteins and glycoproteins (representative image from Movie S1). (B) C6/36 cells infected with nsP3-eYFP/mCherry-E2 dually labeled virus showing the localization of nsP3 around large cytopathic vacuoles (representative image from Movie S2). (C) BHK cells transfected with RNA from nonbudding, dually labeled negative control with _400_YAL_402_/AAA mutation in the E2 (representative image from Movie S3) and nonfusing dually labeled negative control (D) with G_91_D mutation in E1 fusion loop (representative image from Movie S4). (D) Magenta arrows indicate budding viruses, and cyan arrows indicate internalized viruses that are unable to fuse at the endosomes. (E) Live BHK cells infected with nsP2-eYFP/mCherry-E2 virus (green, nsP2-eYFP; red, mCherry-E2) stained with Hoechst stain (nucleus, blue). (F and G) Three-dimensional reconstruction of deconvoluted z-stack images of nsP2-eYFP/mCherry-E2 (F) and nsP3-eYFP/mCherry-E2 (G) virus-infected C6/36 cells. Glycoprotein vesicles are colocalizing with a network of green replication proteins; nsP2 (F [nsP2-eYFP/mCherry-E2]) and nsP3 (G [nsP3-eYFP/mCherry-E2]). (H) Three-dimensional reconstruction of deconvoluted z-stack images of nsP3-eYFP/mCherry-E2 dually labeled virus-infected live BHK and C6/36 (I) cells: green, nsP3-eYFP; red, mCherry-E2; blue, nucleus (Hoechst stain). (J) Live image representing Movie S5 of C6/36 cells infected with mCherry-E2 virus and stained with LysoTracker blue. Green arrows represent LysoTracker blue-positive vesicles, with red glycoprotein-containing endocytic vesicles appearing magenta. (K) Live image representing Movie S6 of C6/36 cells infected with mCherry-E2 virus at 24 h p.i. showing the release of virus from internal cytopathic vacuoles. Virus particles from large cytopathic vacuoles (indicated by green arrows), are transported to the PM and are secreted into the medium by exocytosis. The secreted virus particles stay associated with the filopodial extensions during release (indicated by magenta arrows).

Live imaging of BHK cells infected with the nsP3-eYFP/mCherry-E2 dually labeled virus showed a random distribution of replication and glycoprotein-containing vesicles (Fig. 7A). Nonstructural proteins were segregated and were associated with endosomes and lysosomes. Structural proteins were associated with the membranes in the ER and Golgi pathways, as well as the PM in BHK cells (see Movie S1 in the supplemental material). Replication proteins were present near the PM. C6/36 cells infected with the same dually labeled virus confirmed that the glycoprotein-containing, post-Golgi vesicles (red, CPV-II) rapidly were transported to the PM (see Movie S2 in the supplemental material). These vesicles appeared smaller than the replication protein-associated vesicles and did not contain replication protein nsP3-eYFP (Fig. 7B). Interestingly, endocytic vesicles formed at the PM contained glycoproteins, exhibited rapid retrograde transport toward the larger cytopathic vacuoles with replication proteins, and fused with the latter to form larger stationary vesicles.

10.1128/mBio.02294-16.3 MOVIE S1  BHK cells infected with nsP3-eYFP/mCherry-E2 dually labeled virus showing localization of replication and structural proteins. The replication protein nsP3-eYFP is present on the PM and endosomal and lysosomal vesicles. These vesicles are segregated from mCherry-E2 glycoprotein-containing vesicles. Structural proteins are associated with the membranes in the ER and Golgi pathways, as well as with the PM. In BHK cells, the replication protein nsP3-eYFP is present in cytoplasm and also on the PM, and virus particles bud from the filopodial extension. Download MOVIE S1, AVI file, 12.2 MB. Copyright © 2017 Jose et al. 2017 Jose et al. This content is distributed under the terms of the Creative Commons Attribution 4.0 International license.

10.1128/mBio.02294-16.4 MOVIE S2  Mosquito cells infected with nsP3-eYFP/mCherry-E2 dually labeled virus show colocalization of replication and structural proteins near large cytopathic vesicles. The replication protein nsP3-eYFP is seen arranged on the membrane of large cytopathic vacuoles containing mCherry-E2 glycoproteins. The glycoprotein-containing post-Golgi complex vesicles are rapidly transported to the PM, and endocytic vesicles formed at the PM that contained mature glycoproteins are transported to the larger cytopathic vacuoles associated with replication and fused with the latter to form larger vesicles. Download MOVIE S2, AVI file, 12.9 MB. Copyright © 2017 Jose et al. 2017 Jose et al. This content is distributed under the terms of the Creative Commons Attribution 4.0 International license.

BHK cells transfected with a negative-control, dually labeled (nsP3-eYFP/mCherry-E2), nonbudding cdE2 mutant (_400_YAL_402_/AAA) confirmed the lack of particle budding from filopodial extensions (Fig. 7C), despite glycoprotein transport to the PM and the presence of filopodial extensions (see Movie S3 in the supplemental material). BHK cells transfected with a dually labeled (nsP3-eYFP/mCherry-E2) E1 fusion loop mutant (G91D) demonstrated that particles released from transfected cells can enter uninfected neighboring cells (Fig. 7D) via endocytosis but cannot fuse to the endosomal membranes to initiate a productive infection (see Movie S4 in the supplemental material).

10.1128/mBio.02294-16.5 MOVIE S3  BHK cells transfected with RNA from a nonbudding cdE2 mutant (_400_YAL_402_/AAA) of nsP3-eYFP/mCherry-E2 dually labeled virus. This nonbudding mutant is unable to release fluorescent virus particles from the infected cells due to the absence of a productive CP-cdE2 interaction required for alphavirus budding. The video shows the absence of fluorescent virus particle budding from the PM, even though the PM and filopodial extensions contain mCherry-E2. Download MOVIE S3, AVI file, 5.7 MB. Copyright © 2017 Jose et al. 2017 Jose et al. This content is distributed under the terms of the Creative Commons Attribution 4.0 International license.

10.1128/mBio.02294-16.6 MOVIE S4  BHK cells transfected with RNA from an E1 fusion loop (G91D) mutant of nsP3-eYFP/mCherry-E2 dually labeled virus. This nonfusing mutant produces fluorescent virus particles that are unable to fuse after entering a new cell, where the particles get trapped in the endosome and no virus replication is established postentry, evidenced by the lack of green nsP3-eYFP protein in the newly infected cell even after prolonged imaging. Budding viruses (magenta arrows) and internalized viruses (cyan arrows) that are unable to fuse at the endosomes are marked. Download MOVIE S4, AVI file, 8.4 MB. Copyright © 2017 Jose et al. 2017 Jose et al. This content is distributed under the terms of the Creative Commons Attribution 4.0 International license.

The nsP2-eYFP/mCherry E2 virus-infected cells showed a broader cytoplasmic and nuclear distribution (Fig. 7E) for nsP2-EYFP in BHK cells. Three-dimensional reconstruction of deconvoluted z-stack images of nsP2-eYFP/mCherry-E2 (Fig. 7F) and nsP3-eYFP/mCherry-E2 dually labeled virus-infected live C6/36 cells showed that the replication proteins surrounded the glycoprotein-containing vesicles (Fig. 7G). The glycoproteins were inside the vesicles. The interiors of these large vesicles were remarkably devoid of replication proteins. Three-dimensional reconstruction of deconvoluted z-stack images of nsP3-eYFP/mCherry-E2 dually labeled virus-infected live cells stained with Hoechst stain showed the scattered cytoplasmic distribution of nsP3-eYFP-containing structures in BHK cells (Fig. 7H), whereas these structures were seen associated with the large glycoprotein-containing vesicles in the perinuclear region in C6/36 cells (Fig. 7I). In C6/36 cells infected with mCherry-E2 virus and stained with LysoTracker blue (Fig. 7J), glycoprotein-containing blue vesicles were endocytosed from the PM. These acidic endocytic vesicles fused with larger, preexisting vesicles (labeled in magenta) to form characteristic cytopathic vacuoles (Fig. 7J; see Movie S5 in the supplemental material). The formation of large cytopathic vacuoles was also observed by live imaging in nsP3-eYFP/mCherry-E2 dually labeled virus-infected C6/36 cells stained with DiD and Hoechst stain (see Fig. S2 in the supplemental material). Virus particles were released from the large cytopathic vacuoles, possibly via the secretory pathway (Fig. 7K; see Movie S6 in the supplemental material). This became more prominent in the late stage of virus infection of C6/36 cells. C6/36 cells infected with mCherry-E2 virus at 24 h p.i. showed the release of individual virus particles from the large internal vacuoles. At this stage, the presence of glycoprotein on the PM was drastically reduced (Fig. 7K). The virus particles from the large internal cytopathic vacuoles were transported to the PM and were secreted into the medium by exocytosis. These secreted virus particles stayed associated with the filopodial extensions before getting released into the medium (Movie S6).

10.1128/mBio.02294-16.2 FIG S2  Live image of C6/36 cells infected with nsP3-eYFP/mCherry-E2 virus and stained with DiD (lipid bilayer stain [magenta]) or Hoechst stain (nucleus [blue]), as well as nsP3-eYFP and mCherry-E2 glycoprotein-containing vesicles. A differential interference contrast image of cells collected from transmitted light is also shown (gray). Download FIG S2, TIF file, 2.1 MB. Copyright © 2017 Jose et al. 2017 Jose et al. This content is distributed under the terms of the Creative Commons Attribution 4.0 International license.

10.1128/mBio.02294-16.7 MOVIE S5  Formation of large cytopathic vacuoles in alphavirus-infected mosquito cells after endocytic transport of glycoprotein from the PM. C6/36 cells were infected with mCherry-E2 virus and stained with LysoTracker blue (blue acidic vesicles); glycoprotein-containing vesicles are endocytosed from the PM. These acidic vesicles (magenta, colocalization of blue and red vesicles) are transported to the interior of the cell, where they fuse with larger preexisting vesicles to form the characteristic vesicles containing glycoproteins in the interior of the membrane. Green arrows indicate acidic vesicles moving toward the larger vesicles. These vesicles accumulate internally released fluorescent virus particles as a result of NCs budding through the lipid bilayer of the glycoprotein-containing vesicles from the cytoplasmic side. Download MOVIE S5, AVI file, 9.5 MB. Copyright © 2017 Jose et al. 2017 Jose et al. This content is distributed under the terms of the Creative Commons Attribution 4.0 International license.

10.1128/mBio.02294-16.8 MOVIE S6  Virus release observed from internal cytopathic vesicles. C6/36 cells infected with mCherry-E2 virus at 24 h p.i. show the release of individual virus particles from the large internal vesicles. The virus particles from large cytopathic vesicles are transported (indicated by green arrows) to the PM and are secreted into the medium by exocytosis. The secreted virus particles stay associated with the filopodial extensions before getting released into the media (magenta arrows). Download MOVIE S6, AVI file, 14.3 MB. Copyright © 2017 Jose et al. 2017 Jose et al. This content is distributed under the terms of the Creative Commons Attribution 4.0 International license.

### Characterization of persistently infected C6/36 cells.

**FIG 8:**
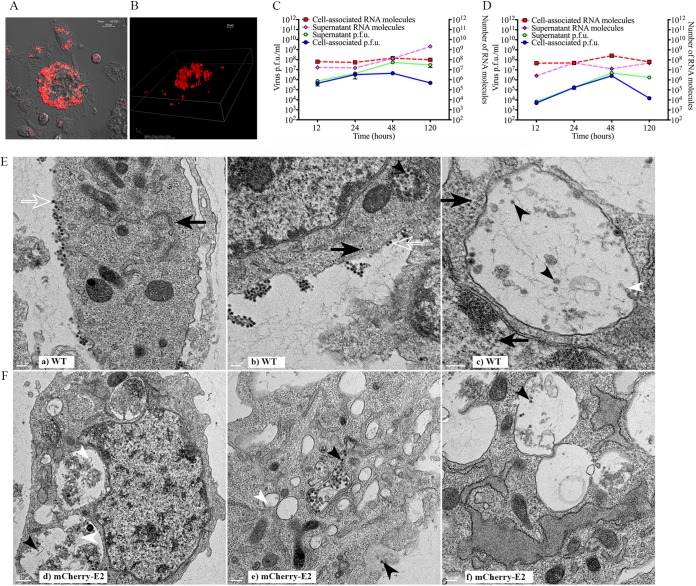
Characterization of persistently infected C6/36 cells. (A) Live image of C6/36 cells persistently infected with mCherry-E2 (a representative image from Movie S7). (B) Three-dimensional reconstruction of deconvoluted z-stack images showing the presence of mCherry-E2 glycoprotein-containing large cytopathic vacuoles in C6/36 cells persistently infected FP-tagged virus. (C and D) Quantification of number of RNA molecules and PFU in C6/36 cells persistently infected with WT (C) or mCherry-E2 (D) virus. C6/36 cells persistently infected with WT virus were plated onto 35-mm culture dishes (10^6^ cells/ml), and virus PFU and RNA were collected from cell culture supernatants and lysed cells at the indicated time points postplating. (E and F) TEM analysis of C6/36 cells persistently infected with WT (Ea to -c) and mCherry-E2 (Fd to -f) virus. Budding virus (open white arrows), budded virions (solid black arrowheads), NCs (solid black arrows), and replication spherules (solid white arrowheads) are shown. Scale bars represent 200 nm for panels Ea to -c and Fb and -c. For panel Fd, the scale bar represents 500 nm. Cytopathic vacuoles that contain internally budded particles are lined with NCs on the cytoplasmic side (Eb and Fd). Budded virus particles associate with the filopodial extension outside the cell similar to acute infection (Eb).

C6/36 cells were passaged in the presence of WT SINV or mCherry-E2-tagged virus for 30 days, and persistently infected cell lines were generated for live-cell imaging, thin-section TEM analysis, qRT-PCR, and plaque assays. The imaging results showed that the mCherry-E2 glycoprotein was present exclusively in large cytopathic vacuoles and was completely absent on the PM and filopodial extensions (Fig. 8A and B; see Movie S7 in the supplemental material). Quantification of RNA molecules and PFU from C6/36 cells persistently infected with WT SINV (Fig. 8C) and mCherry-E2-tagged viruses (Fig. 8D) showed that overall virus replication and particle production were significantly reduced. A time course experiment with persistently infected cells (at 12, 24, 48, and 120 h) revealed that virus replication in the persistently infected cells remains steady, virus particles released into the medium accumulate in supernatant, and the number of virus particles increases with time. Thin sections of C6/36 cells (Fig. 8E and F) showed that persistently infected cells contained internally budded virions (solid black arrowhead), NCs (solid black arrow), budding virus (open white arrow), and replication spherules (white arrowhead) similar to those seen in acutely infected cells, although the quantities of each of them were significantly reduced. Cytopathic vacuoles that contained internally budded particles were observed that were lined with NCs on the cytoplasmic side (Fig. 8Eb). The mCherry-E2 mutant virus was included in the characterization of replication and budding experiments to corroborate the results obtained from live imaging studies. Live imaging data pertaining to the site of virus assembly were derived from the mCherry-E2-tagged rather than WT virus.

10.1128/mBio.02294-16.9 MOVIE S7  C6/36 cells persistently infected with mCherry-E2 virus show the absence of mCherry-E2 protein at the PM and filopodial extensions. mCherry-E2 is present only on the large cytopathic vesicles and does not accumulate glycoproteins on the PM. The virus budding frequency is greatly reduced, and virus assembly and budding are predominantly associated with the internal, large glycoprotein-containing cytopathic vacuoles. Download MOVIE S7, AVI file, 8.7 MB. Copyright © 2017 Jose et al. 2017 Jose et al. This content is distributed under the terms of the Creative Commons Attribution 4.0 International license.

## DISCUSSION

Single-particle tracking and real-time live imaging provide powerful tools for obtaining high-quality spatial and temporal resolution. This contrasts with traditional modes of TEM and superresolution light microscopy that provide high spatial but no temporal resolution. In this study, we used live imaging coupled with fluorescent protein (FP)-tagged viral proteins to analyze spatial and temporal aspects of alphavirus infection in mammalian host and mosquito vector cells. We generated dually labeled FP-tagged viruses with eYFP tagged to either nsP2 or nsP3 (11, 12) and mCherry fused to the N terminus of the E2 glycoprotein, which is known to tolerate insertions of immunoglobulin-binding domains of protein L (18) and fluorescent proteins (13–15, 17, 19).

We analyzed virus replication and assembly simultaneously using dually labeled virus-infected BHK and mosquito cells, where CPV-I and CPV-II were labeled separately with nsP3-eYFP and mCherry-E2, respectively. The RCs are composed of all four nonstructural proteins (nsP1 to -4) along with host factors and viral RNA (20). In BHK cells, we observed localization of the nonstructural proteins to the PM and internal vesicles, and this result was confirmed by TEM and IF analysis. Only nsP1 was localized to the filopodial extensions, which emphasizes the role of nsP1 in transport of replication complexes to the PM and host actin modification. Structurally distinct CPV-I and CPV-II were detected in SINV-infected BHK cells, but not in infected mosquito cells, which suggests that the strategy for virus replication in mosquito cells utilizes a spatially distinct set of membranous structures. The spherules on CPV-I provide the attachment sites for nonstructural proteins and viral RNA, enhance replication efficiency by concentrating the replicative enzymes (6), and make the viral RNA replication intermediates inaccessible to the host cell’s antiviral systems (8). Our observations suggest that the NCs assemble near CPV-I, and nascent RNA may be extruded from the spherules followed by coassembly with CP. In both BHK and mosquito cells, NCs were seen associated with the PM, which suggests the interaction of cdE2 with NCs (21). Furthermore, NCs were found attached to CPV-II in BHK and large cytopathic vesicles in mosquito cells. However, in mosquito cells, with the exception of nsP1 that localized to the filopodial extension, the replication proteins (nsP2 to -4) and dsRNA were not detected at the PM by IF. The localization of nsP1 to the filopodial extensions in C6/36 cells was similar to that in BHK cells. In mosquito cells, we observed large (~2-μm-diameter) vesicles associated with replication spherules that are similar to CPV-I (0.5-μm diameter). NCs were also found attached to these large vesicles in C6/36 cells, whereas in BHK cells, NCs were attached to CPV-II (0.2-μm diameter). In addition, vesicles (0.6-μm diameter) that enclose >50 virus particles were observed in mosquito but not BHK cells. The TEM analyses provided spatial resolution of the virus RCs observed by live imaging and also confirmed that the sites of virus replication and assembly were not segregated in mosquito cells. This vital observation was corroborated by the absence of replication spherules at the PM of mosquito cells, as observed by the TEM analysis.

Live imaging was used to track the motility of glycoprotein-containing vesicles in BHK cells. TEM analysis suggested that CPV-II traffics to the PM via the secretory pathway. Microtubule-associated core trafficking was implicated in hepatitis C virus, which was consistent with secretory vesicle trafficking (22). Our data suggest that NCs that assemble near CPV-I traffic to the PM, with a few of them associated with CPV-II and the rest trafficked by unknown host transport machinery. NCs then interact with glycoprotein spikes at the PM to form virions. By utilizing live imaging and TEM, we uncovered a novel mechanism of alphavirus infection in mosquito cells, where virus budding occurs initially at the PM, but at ~9 h p.i., internalization of surface glycoproteins generates vesicles that traffic to the perinuclear, large cytopathic vacuoles. These large cytopathic vacuoles contain glycoprotein in the inner membrane and replication proteins and dsRNA outside the limiting membrane. NCs were also found on the outer membrane of these vacuoles by TEM analysis. As the virus assembly sites are established on the membranes of these large vesicles, virus budding occurs internally, and the budded virions utilize the secretory pathway for egress. We have also observed the direct transport of budded virions to uninfected cells in dually labeled virus-infected C6/36 cells. The direct cell-to-cell transport of single particles bypassing the extracellular medium increases virus dissemination and may be involved in establishing persistent infection (23).

After an initial, acute phase of virus production, alphavirus infection in mosquito cells becomes persistent and noncytopathic (1), which is necessary for arboviruses to be continuously transmitted in nature. Mosquito cells that were persistently infected with mCherry-E2 virus showed no accumulation of glycoproteins on the PM, and virus assembly and budding were associated predominantly with the large, glycoprotein-containing, cytopathic vacuoles. Furthermore, compared to acute infections, we detected fewer replication spherules and internally budded virus particles among intraluminal vesicles in the large cytopathic vacuoles, suggesting that lysosomal fusion and degradation of contents had occurred. Although our growth kinetic experiments suggested the presence of virus particles both inside and outside cells, there was an overall 3-log reduction in virus replication and virus particle production compared to normal SINV infection of C6/36 cells. Based on these observations and the data obtained from our mosquito cell studies, we hypothesize that alphaviruses counteract the host response by altering the site of replication and assembly and by using lysosomal degradation of virions to reduce the population of internally budded, cell-associated virus.

**FIG 9:**
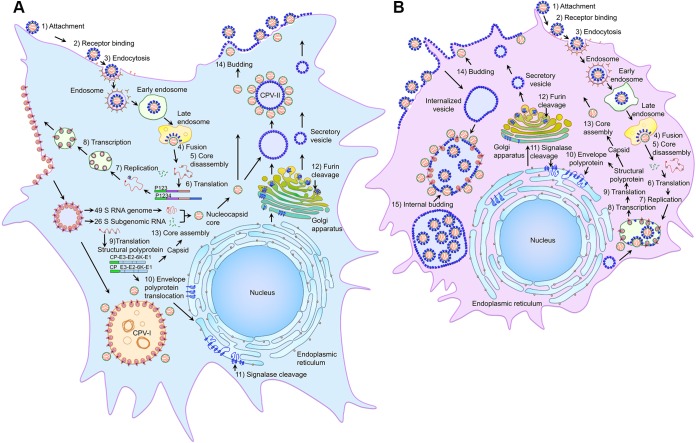
Models of the alphavirus life cycle and the virus-induced structures in mammalian (A) and insect (B) cells. Following attachment and receptor binding (steps 1 and 2), SINV is internalized by clathrin-mediated endocytosis (step 3). Low-pH-mediated fusion (step 4) in the late endosome releases nucleocapsid (NC), and after disassembly (step 5), nonstructural polyproteins are translated (step 6) from viral mRNA. Replication proteins and host proteins along with the viral RNA form replication complexes (step 7) that replicate and transcribe (step 8) viral RNA and induce spherule structures on endosomal and plasma membranes. Internalization of replication spherules from the plasma membrane via vesicles and subsequent fusion of these vesicles with lysosomes generate CPV-I structures. Structural polyprotein translated from the subgenomic RNA (step 9) is processed into capsid protein (CP), and envelope polyproteins that are translocated (step 10) to the ER, processed by signalase (step 11) and glycosylated and transported through the Golgi complex, where fuin cleavage removes E3 from E2 (step 12) to the PM via the secretory pathway. CP binds genome RNA to form NC (step 13) that binds the glycoprotein spikes on the PM and virus buds from PM (step 14). CPV-II structures that contain glycoprotein spikes and attached NCs originate from the Golgi complex and accumulate in the cells at the late stage of infection. In mosquito cells, replication spherules are present only on large internal vesicles that also contain NCs and internally budded virus particles. Glycoprotein-containing vesicles are internalized from the plasma membrane, and fusion of smaller internalized vesicles produces large cytopathic vacuoles. Virus particles bud into the large cytopathic vacuoles (step 15), which also contain replication spherules and nucleocapsid cores. Internally budded virions accumulate in large vesicles. Spherules on the plasma membrane and structurally distinct CPV-I and CPV-II structures are not observed in mosquito cells.

Based on our live-cell imaging experiments complemented by TEM and IF, we propose a model (Fig. 9) for spatial and temporal regulation of the alphavirus life cycle in mammalian host and insect vector cells. In mammalian cells (Fig. 9A), the nonstructural polyprotein translated from the viral RNA, presumably in association with endosomal membrane, is directed to the inner leaflet of the PM by nsP1. At the PM, the replication proteins along with host proteins form an RC and induce the production of spherules and dsRNA. Following endocytosis, the spherules on the endosomal vesicles are transferred to the membranes of large vesicles in the cytoplasm and form CPV-I, presumably after the endosomes fuse with lysosomes. However, in mosquito cells (Fig. 9B), the RC established on the large endolysosomal membrane is not transported to the PM. Maximal virus replication coincides with the formation of RCs on CPV-I. In both mammalian and insect cells, the endocytic and secretory pathways play important roles in virus replication and assembly by transporting CPV-I and CPV-II to the PM, as demonstrated in the live imaging experiments. Consistent with our previous findings (21, 24), NCs that interact with the cell surface glycoproteins generate virions that utilize the filopodial extensions for viral dissemination while averting superinfection. In mammalian cells, the viral glycoproteins are segregated from the replication proteins (CPV-I) and reside in the membranes of the ER, Golgi complex, CPV-II, and PM, and virus buds from the PM. However, in mosquito cells, the viral glycoproteins and the virus budding process occur at the PM as well as the internal vesicles. We observed there was concomitant internalization of glycoprotein from the PM at ~9 h p.i. in mosquito cells. We report the spatial and temporal dynamics of viral proteins by live imaging, although the entire model for alphavirus infection of mosquitos may not be represented by C6/36 cells that are known to have a dysfunctional RNA interference (RNAi) system (25). Many of the players and molecular mechanisms involved in these complex processes causing such drastic changes in mosquito vectors remain to be established. We hypothesize that in mosquito vectors, alphavirus replication and assembly machineries have evolved to favor transition to a state of persistent infection, thus enabling these highly efficient viruses to thrive in nature.

## MATERIALS AND METHODS

### Viruses and cells.

The fluorescent protein (FP)-tagged SINV constructs were generated using pToto64, a full-length cDNA clone of SINV (26), using standard overlapping PCR mutagenesis. BHK-15 cells were obtained from the American Type Culture Collection (ATCC) and were maintained in minimal essential medium (MEM) (27) supplemented with 10% fetal bovine serum (FBS). Mosquito (*Aedes albopictus*) C6/36 cells (ATCC) were maintained at 30°C in the presence of 5% CO_2_ in MEM supplemented with 2 mM l-glutamine, 0.1 mM nonessential amino acids, 25 mM HEPES, and 10% heat-inactivated FBS. Viruses were propagated in BHK-15 cells at 37°C and C6/36 cells at 30°C in MEM supplemented with 5% FBS in the presence of 5% CO_2_ unless otherwise noted. Persistently infected mosquito cells were generated after serial passaging of the WT SINV or FP-tagged SINV-infected C6/36 cells for 30 days.

### Construction of FP-tagged viruses.

The dually labeled nsP2-eYFP/mCherry-E2 construct was generated by subcloning an AfeI-ClaI insert from the nsP2-eYFP plasmid into the mCherry-E2 plasmid vector, a SINV cDNA clone with N-terminal mCherry tag on E2 (15). Similarly, the nsP3-eYFP/mCherry-E2 dually labeled virus was generated by cloning the nsP3-eYFP insert using the AvrII-PasI restriction sites into the mCherry-E2 plasmid. Previously characterized cdE2 mutations (_400_YAL_402_/AAA) (21) and an E1 (G91D) fusion loop mutation (28) were generated by overlapping PCR and were cloned into the mCherry-E2 plasmid and nsP3-eYFP/mCherry-E2 dually labeled plasmid using BssHII-BsiWI restriction sites.

### *In vitro* transcription and transfection.

The wild-type (WT) and FP-tagged cDNA clones were linearized with SacI and *in vitro* transcribed with SP6 RNA polymerase, transfected into BHK-15 cells by electroporation as previously described (21). Virus released from the transfected cells was quantified by standard plaque assay using supernatant collected from the transfected cells at 24 h postelectroporation. Virus stocks were generated after passaging a single plaque for two passages in BHK-15 cells. Total RNA was extracted from infected BHK-15 cells using the RNeasy kit (Qiagen, Valencia, CA) and was used for reverse transcription (RT)-PCR using the cMaster RT-PCR kit (Eppendorf, Inc., Westbury, NY). The presence of tags and mutations in each virus was confirmed by sequencing the RT-PCR products corresponding to the nsP2, nsP3, E2, and E1 coding regions.

### Growth kinetic analyses.

One-step growth analyses were performed as described previously to measure growth kinetics of the FP-tagged viruses (21). BHK-15 and C6/36 cells, seeded into 35-mm culture dishes, were infected with virus at a multiplicity of infection (MOI) of 5 for 1 h at room temperature. Infected cells were washed extensively with MEM and incubated further in MEM supplemented with 5% FBS at 37°C for BHK-15 and 30°C for C6/36 cells in the presence of 5% CO_2_. Virus supernatants were collected, and medium over cells was replaced with fresh medium every hour for 12 h postinfection. To determine the cumulative growth kinetics, cells in separate 35-mm culture dishes were infected as described elsewhere, and at the indicated time points (every hour for the first 12 h and then at 18 h and 24 h), infected cells and culture media were harvested. The cells were washed three times with phosphate-buffered saline (PBS), and cell-associated viruses were isolated by the freeze-thaw method in PBS containing 1% FBS, 10 mM CaCl_2_, and 10 mM MgCl_2_. To analyze the virus growth in persistently infected C6/36 cells, 10^6^/ml C6/36 cells persistently infected with WT SINV were plated on 35-mm-diameter culture dishes, and at the indicated time points (12, 24, 48, and 120 h postplating), the cells and culture medium were harvested. Plaque-forming virus from each sample of virus supernatant and cell-associated extracts was assayed by titration on BHK cell monolayers at 37°C. All experiments were conducted in triplicate.

### qRT-PCR.

The numbers of virus particles released at different time points and total RNA molecules in infected cells and persistently infected C6/36 cells were determined by qRT-PCR as previously described (29). RNA was extracted either from virus supernatants or cytoplasmic extracts using the RNeasy kit (Qiagen, Valencia, CA) according to the manufacturer’s instructions. qRT-PCR was done using the primers 5′ TTCCCTGTGTGCACGTACAT 3′ and 5′ TGAGCCCAACCAGAAGTTTT 3′, which bind nucleotides 1044 to 1063 and nucleotides 1130 to 1149 of the SINV genome, respectively. qRT-PCRs were performed using the SuperScript III Platinum SYBR green one-step qRT-PCR kit (Invitrogen, Grand Island, NY) in triplicate in 25-μl sample volumes that contained a 5-μl aliquot of purified viral RNA. The cycling parameters were 4 min at 50°C and 5 min at 95°C, followed by 40 cycles of 5 s at 95°C and 1 min at 60°C. The viral RNA copy number was determined using a standard curve of the cycle threshold (*C*_*T*_) values determined by qRT-PCR versus the number of molecules of *in vitro*-transcribed genomic RNA using the primers.

### Determination of specific infectivity of FP-tagged viruses.

Confluent BHK-15 cells in 35-mm culture dishes were infected with WT, nsP3-eYFP, mCherry-E2, or the dually labeled nsP3-eYFP/mCherry-E2 virus at an MOI of 2 for 1 h at room temperature. Cells were incubated at 37°C for 12 h. Virus supernatants were collected at 4, 6, 10, and 12 h p.i. Viral RNA copy number was determined by qRT-PCR, and the number of PFU was determined for the same sample by plaque assays on BHK-15 cells. These experiments were conducted in triplicate. The specific infectivity was calculated for each time point from the number of RNA molecules over PFU.

### Analysis of nonstructural and structural polyprotein processing.

Expression and processing of nonstructural and structural polyproteins were characterized by Western immunoblot analysis of lysates of infected BHK-15 cells. Cells from 35-mm plates were lysed using TNE buffer (25 mM Tris-HCl [pH 7.4], 100 mM NaCl, 5 mM EDTA) with 1% Triton X-100 at 12 h p.i., and proteins were separated on a 10% Bis-Tris precast SDS-PAGE gel (Bio-Rad), electrophoretically transferred to nitrocellulose membranes, and probed either separately or in combination with SINV-specific rabbit polyclonal anti-nsP2, anti-nsP4, anti-E2 (a gift from J. H. Strauss), and anti-CP antibodies as described previously (21). Infrared-labeled (IRDye 680 and IRDye 800 goat anti-rabbit and goat anti-mouse secondary antibodies [LI-COR]) were used to detect proteins, and the blots were scanned with an Odyssey infrared imager (LI-COR, Lincoln, NE), using Odyssey software version 3.

### IF assay.

IF assays were performed on BHK-15 and C6/36 cells grown on glass coverslips. The primary antibodies used in the experiments were SINV-specific rabbit polyclonal anti-nsP1 and anti-nsP4. The dsRNA molecules were detected using mouse anti-dsRNA antibody (English and Scientific Consulting, Szirak, Hungary). For IF, infected cells were fixed using 3.7% paraformaldehyde for 15 min at room temperature and permeabilized using 0.1% Triton X-100 in phosphate-buffered saline (PBS) for 5 min. The secondary antibodies used were fluorescein isothiocyanate (FITC) or tetramethyl rhodamine isothiocyanate (TRITC)-conjugated goat anti-rabbit and goat anti-mouse antibodies (Pierce) in PBS with 10 mg/ml bovine serum albumin. Actin filaments were stained using Alexa Fluor 488 phalloidin (Invitrogen), and nuclei were stained using Hoechst stain (Invitrogen) according to the manufacturer’s instructions. Images were acquired using a Nikon A1R-MP confocal microscope at room temperature with a 60× oil objective and 1.4 normal aperture (NA). Images were processed using the NIS Elements software (Nikon), and the brightness and contrast were adjusted using nonlinear lookup tables.

### Live-cell imaging.

Live-cell imaging was conducted as described previously (15). Briefly, BHK-15 or C6/36 cells were seeded onto a 4-chamber borosilicate cover glass (Fischer Scientific Pittsburgh, PA) and infected with fluorescent virus at an MOI of 50 at 25% confluence. Infected cells were imaged after media were replaced with Opti-MEM I reduced-serum medium (Invitrogen). Live-imaging-compatible stains were obtained from Invitrogen/Molecular Probes. These included Hoechst stain (nucleus), Vybrant DiD cell-labeling solution (membrane vesicles), and LysoTracker blue (acidic vesicles), which were used in conjunction with FP-tagged viruses. Live imaging was conducted using a heated 60× oil immersion objective (1.4 NA) in a live imaging chamber (Tokai Hit, Fujinomiya, Shizuoka Prefecture, Japan) supplied with 5% CO_2_ at 37°C for BHK-15 cells and 30°C for C6/36 cells. The lasers and emission band-passes used for imaging were as follows: blue, excitation of 405 nm and emission of 425 to 475 nm; green, excitation of 488 nm and emission of 500 to 550 nm; red, excitation of 561 nm and emission of 570 to 620 nm; far red, excitation of 640 and emission of 660 to 740 nm. Differential interference contrast images were also collected from transmitted light along with fluorescent images for colocalization of viral proteins in the cellular organelles. NIS-Elements software was used for image acquisition and analysis. Images were collected for 10 min, and time-lapse videos were generated from the acquired images. To generate videos, live images were collected at frame rates ranging from 0.8 to 1 frames per s (fps) for a time scale of 1 to 30 min, and time-lapse videos were generated from the acquired images at a frame rate of 5 to 7 fps using ImageJ (NIH, Bethesda, MD, USA).

### Thin-section TEM.

BHK-15 and C6/36 cells infected with wild-type or mCherry-E2-tagged SINV at an MOI of 5 were fixed at 6, 12, or 24 h p.i. Persistently infected C6/36 cells were fixed at 48 h after seeding. Cells were fixed for 3 days in 2.5% glutaraldehyde in 0.1 M sodium cacodylate buffer, embedded in 2% agarose, postfixed for 90 min in buffered 1% osmium tetroxide containing 0.8% potassium ferricyanide, and stained for 45 min in 2% uranyl acetate. They were then dehydrated with a graded series of ethanol, transferred into propylene oxide, and embedded in EMbed-812 resin. Thin sections were cut on a Reichert-Jung Ultracut E ultramicrotome and stained with 2% uranyl acetate and lead citrate (30). Images were acquired in an FEI Tecnai G^2^ 20 electron microscope equipped with a LaB_6_ source and operated at 100 keV (Life Science Microscopy Facility, Purdue University).
