# Supplementary figures and images for: Spatial and Temporal Analysis of Alphavirus Replication and Assembly in Mammalian and Mosquito Cells
Source: mBio. 2017 Feb 14;8(1):e02294-16. doi: 10.1128/mBio.02294-16 (PMC5312085; doi:10.1128/mBio.02294-16)

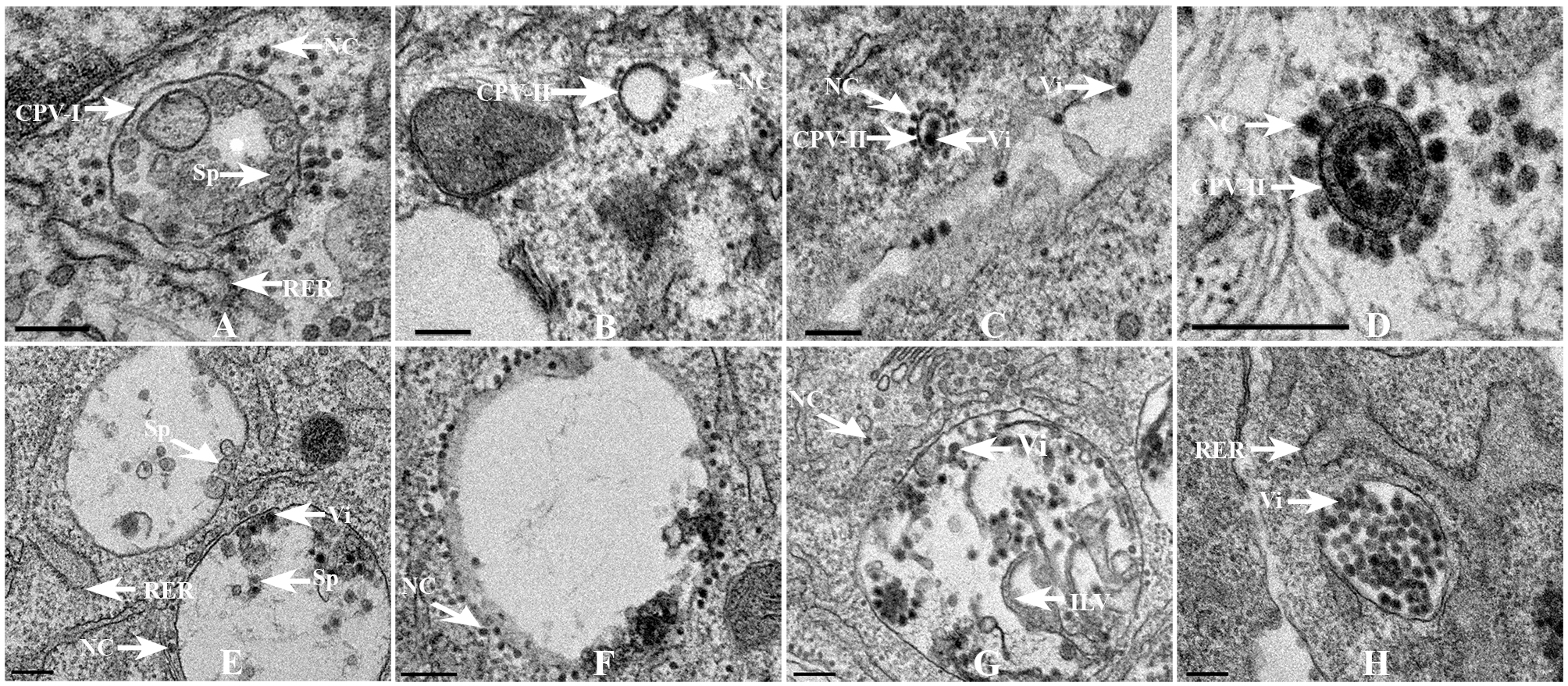

Supplement: FIG S1 [file mbo001173177sf1.tif]

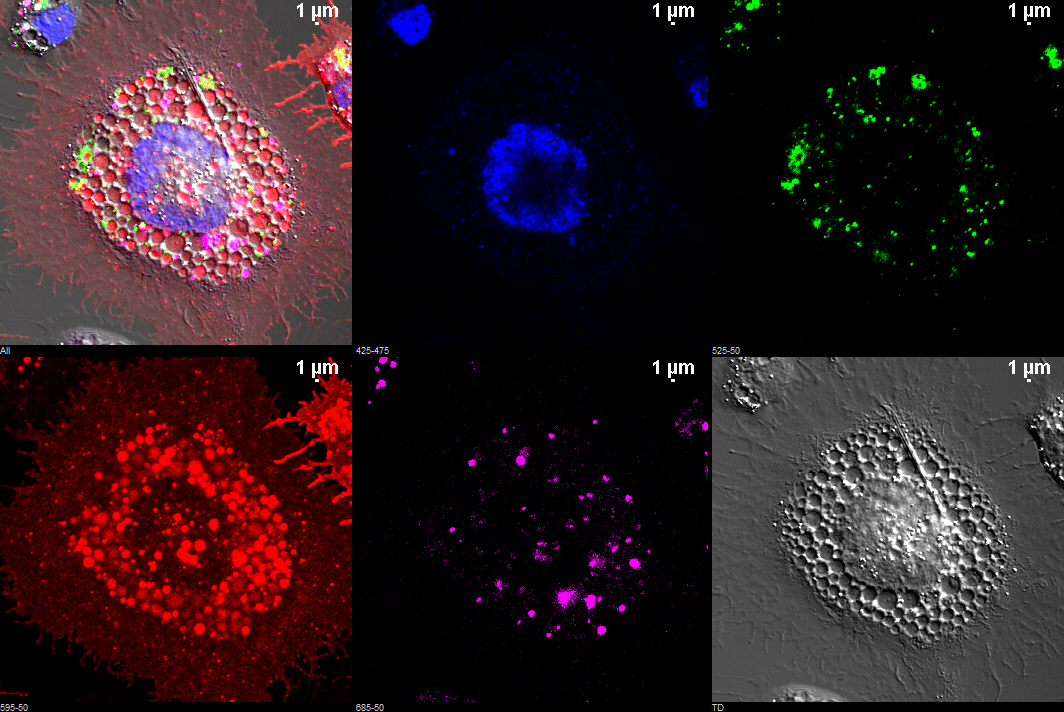

Supplement: FIG S2 [file mbo001173177sf2.tif]
